# Ubiquitin crosstalk connecting cellular processes

**DOI:** 10.1186/1747-1028-1-21

**Published:** 2006-09-28

**Authors:** Tom AM Groothuis, Nico P Dantuma, Jacques Neefjes, Florian A Salomons

**Affiliations:** 1 Department of Cell and Molecular Biology, The Medical Nobel Institute, Karolinska Institutet, Von Eulers väg 3, S-17177, Stockholm, Sweden; 2 Division of Tumor Biology, The Netherlands Cancer Institute, Plesmanlaan 121, 1066 CX, Amsterdam, The Netherlands

## Abstract

The polypeptide ubiquitin is used in many processes as different as endocytosis, multivesicular body formation, and regulation of gene transcription. Conjugation of a single ubiquitin moiety is typically used in these processes. A polymer of ubiquitin moieties is required for tagging proteins for proteasomal degradation. Besides its role in protein degradation, ubiquitin is also engaged as mono- or polymer in intracellular signalling and DNA repair. Since free ubiquitin is present in limiting amounts in cells, changes in the demands for ubiquitin in any of these processes is likely to indirectly affect other ubiquitin modifications. For example, proteotoxic stress strongly increases poly-ubiquitylated proteins at the cost of mono-ubiquitylated histones resulting in chromatin remodelling and altered transcription. Here we discuss the interconnection between ubiquitin-dependent processes and speculate on the functional significance of the ubiquitin equilibrium as a signalling route translating cellular stress into molecular responses.

## Background

**Figure 1:**
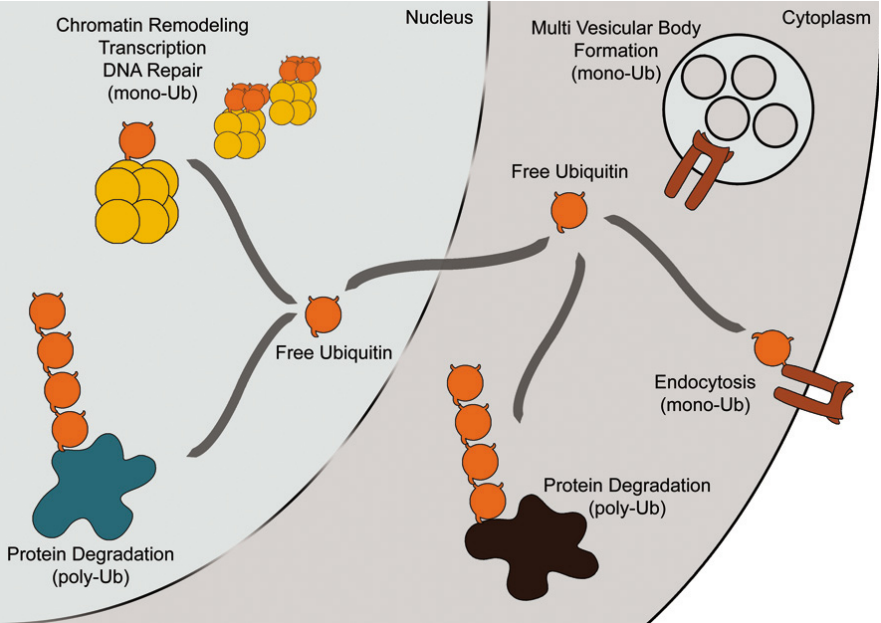
**Ubiquitin: forms and functions**. Free ubiquitin molecules are present in both the nucleus and the cytosol; the protein is small enough for passive diffusion through the nuclear pore between the two compartments. Ubiquitin conjugation to target proteins plays a central role in many processes of the cell. The best-known function of ubiquitin is its involvement in protein degradation via poly-ubiquitylation in the nucleus and cytoplasm. Mono-ubiquitylation of proteins has various functions depending on the target protein; it can vary from involvement in endocytosis at the plasma membrane, to DNA repair in the nucleus.

Ubiquitin is a small polypeptide (76 amino acids) used in many essential cellular processes. Ubiquitin is abundantly expressed in eukaryotes and can be found in all cell types and tissues with up to 10^8 ^copies per cell [1]. Processes as different as endocytosis, signal transduction, DNA repair, transcription and chromatin remodelling require ubiquitin for proper functioning (reviewed in [2-6]; Figure 1). Biochemical studies suggest that a polymer of four or more ubiquitin moieties is required to label protein substrates for recognition by proteasomes [7,8]. Ubiquitin is post-translational conjugated to protein substrates through an isopeptide bond between the C-terminal glycine residue of ubiquitin and the ε-amino group of a lysine residue or sometimes the α-amino group of a target protein. The conjugated ubiquitin can be a substrate for further ubiquitylation through one of its seven lysine residues leading to the formation of a poly-ubiquitin chain. Single ubiquitin and poly-ubiquitin conjugates can be recognized by various proteins containing ubiquitin binding domains (UBDs). These UBDs act similar as, for example, SH2 and SH3 domains that bind their targets dependent on phosphorylation of specific target residues. These post-translational modifications are a general mechanism for regulating protein interactions [9]. A large number of different UBDs have been identified in unrelated proteins underscoring the complexity and versatility of ubiquitin modifications and ubiquitin-dependent interactions.

Ubiquitin contains seven lysine residues, all of which can be used to form poly-ubiquitin chains [10]. Poly-ubiquitin chains linked through lysine-48 are most common and usually target substrate proteins for proteolysis. Other ubiquitin modifications, like poly-ubiquitylation through lysine-6 and lysine-63 are used for processes like DNA repair, endocytosis, and ribosomal protein synthesis [11-15]. Mono-ubiquitylation is involved in endocytosis, multivesicular body formation and chromatin remodelling [16]. As a major constituent of chromatin, histones are subjected to several post-translational modifications including ubiquitylation [17,18]. Ubiquitylation of histones affect transcriptional activity and chromatin remodelling [4,19] and has recently been reported to be involved in DNA repair mechanisms as well [20-22].

A cascade of different classes of enzymes is required for identification and ubiquitylation of proteins (reviewed in [23,24]). The first step in ubiquitylation is performed by the E1 ubiquitin-activating enzyme, which activates ubiquitin by formation of a thiol-ester bond between a cysteine residue of E1 and the carboxyl terminus of ubiquitin [25]. The activated ubiquitin molecule is subsequently passed on to one of the different E2 ubiquitin conjugating enzymes, which also establishes a thiol-ester linkage with ubiquitin. Substrate proteins are recognized by a specific E3 ubiquitin ligase, which, in combination with E2 enzymes, ubiquitylate the substrate [26]. Combinations of about twenty human E2 conjugating enzymes with several hundreds of distinct E3 ubiquitin ligases enlarge the variety and specificity in recognizing and ubiquitylating target proteins. Similar to most post-translational signalling modifications, ubiquitin modifications are dynamic. Ubiquitin can be removed from substrates by a heterogeneous family of specific deubiquitylation enzymes (DUBs) [27]. DUBs are proteases that catalyze the cleavage between the C-terminal glycine-76 of ubiquitin and the substrate. DUBs may thus counteract specific processes by removing mono-ubiquitin or poly-ubiquitin from various substrates like histones, proteasome substrates and other proteins. For example, the 19S lid of the proteasome contains a DUB (Rpn11) for the removal of poly-ubiquitin from proteasome substrates prior to proteolysis [28,29]. In addition to deubiquitylation activities, DUBs are involved in processing newly synthesized, inactive ubiquitin precursors. Thus DUBs generate all free ubiquitin molecules, and are essential for the progression of the ubiquitin cycle and the (re)generation of non-conjugated, free ubiquitin, which can be used for new ubiquitylation reactions. Here we discuss the ubiquitin homeostasis and its link to various cellular processes.

## Different pools of ubiquitin

Several groups have described the existence of various forms of ubiquitin in eukaryotic cells, including free ubiquitin molecules, mono- and poly-ubiquitylated proteins [30-34]. These pools are not static and ubiquitin cycles dynamically between these pools mediated by ubiquitylation and deubiquitylation enzymes [35,36]. The dynamics of the different ubiquitin pools could be visualized in living cells using a GFP-ubiquitin (GFP-Ub) fusion construct [37]. Although this construct is about 4-fold larger than unmodified ubiquitin, it still reflected in many aspects the behaviour and localization of the endogenous protein in mono-ubiquitin modification and use in poly-ubiquitin chains for degradation [37,38]. The majority of ubiquitin is present in (large) conjugates while only a small fraction is free. A major pool of ubiquitin is conjugated to histone 2A and 2B under normal circumstances [4,37].

To monitor the amount of free ubiquitin in living cells the nuclear pore was used as a molecular sieve in a FLIP (Fluorescence Loss In Photobleaching) protocol wherein the GFP fluorescence in either the complete nucleus or cytoplasm was bleached and the effect of GFP-Ub fluorescence in the other compartment was measured. The rationale behind this approach is that proteins up to approximately 50 kDa can passively diffuse through the nuclear pore, whereas larger species (like conjugated ubiquitin) are excluded [39]. The FLIP experiment revealed different pools of GFP-Ub in the cytosol as well as the nucleus. A small fraction of GFP-Ub rapidly diffused from the non-bleached into the bleached compartment, representing the free pool of unconjugated GFP-Ub. Slowly other GFP-Ub entered the bleached compartment that may have resulted from generation of free GFP-Ub by release from substrate proteins like histones and proteasome substrates by DUBs. Similar results were obtained with a photoactivatable form of GFP-Ub where a region in the nucleus was activated and fluorescence accumulated slowly in the cytoplasm in time. These observations indicate that during physiological conditions only a small portion of ubiquitin is in the monomeric form. Using these approaches, three distinct ubiquitin pools could be distinguished in the cell: a small fraction of free monomeric ubiquitin; a major fraction of ubiquitylated proteins and mono-ubiquitylated histones. Smaller amounts of ubiquitin are used for processes like endocytosis and multivesicular body formation and therefore not easily detected by live cell imaging where only the major fractions of ubiquitin are distinguishable.

## Ubiquitin homeostasis

**Figure 2:**
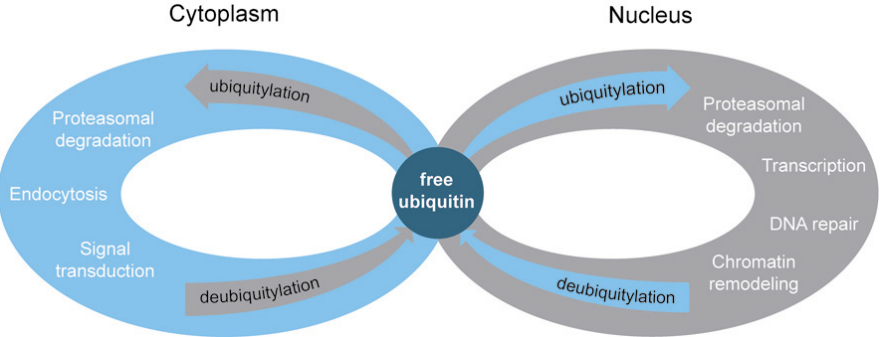
**The ubiquitin cycle**. Free ubiquitin plays a central role in the biochemistry of the cell; all processes that consume ubiquitin ultimately have to derive it from freely available ubiquitin. Because the amount of free ubiquitin is relatively small, processes that consume large amounts of ubiquitin will indirectly influence other cellular processes that depend on ubiquitin.

Given the availability of a small pool of free ubiquitin, free ubiquitin has to be replenished continuously by DUBs. This ubiquitin cycle is essential to supply ubiquitin to substrates in a multitude of nuclear and cytosolic processes (Figure 2). But what happens when the ubiquitin equilibrium is disturbed? Inhibition of the proteasome results in accumulation of poly-ubiquitylated proteins. This is a reflection of proteotoxic stress since identical effects on poly-ubiquitin were observed following cell exposure to thermal stress conditions. Under these conditions, heat-labile proteins denature and provide the cell with an overload of proteasomal substrates. After a heat shock, the quantity of poly-ubiquitylated proteins increased dramatically. Since free ubiquitin is present in only in limiting amounts and neo-synthesis cannot compensate the acute needs for ubiquitin, this implies that ubiquitin molecules have to come from other sources to accommodate the increase in poly-ubiquitylated species. Accordingly several studies have shown that, following proteotoxic stress by proteasome inhibition, a redistribution of ubiquitin from the nucleus to the cytosol was observed in parallel with deubiquitylation of histones [37,40,41].

In principle, histone deubiquitylation could be the result of enhanced deubiquitylation activity following proteotoxic stress. This was assayed using photo-activated GFP-Ub in a protocol where the fate (i.e. the off-rate) of ubiquitin fluorescence was followed in one half of the nucleus. Proteotoxic stress did not affect the off-rate of fluorescent (photoactivated) GFP-Ub from histones indicating the DUBs were not activated by proteotoxic stress [37]. Antibody microinjection experiments supported the idea that histone deubiquitylation was the result of an altered equilibrium in the ubiquitin cycle [37]. Proteotoxic stress results in an increased requirement for free ubiquitin for incorporation in poly-ubiquitylated substrates at the cost of mono-ubiquitylated histones. We speculate that through the limited pool of ubiquitin enhanced poly-ubiquitylation following proteotoxic stress is sensed by the nucleus by affecting the histone-ubiquitin status and thus the transcriptome.

## Coupling cellular processes by the ubiquitin cycle

Ubiquitylation of histones is one of the major (and largest) modifications in chromatin. This modification is in mammalian cells mainly found on the core histone H2A. Approximately 5–15% of histone H2A is ubiquitylated, and this is associated with condensed DNA and gene silencing [42-44]. H2B ubiquitylation is essential for the sliding activity of RNA polymerase II and regulates transcription [45]. Deubiquitylation of histones during proteotoxic stress conditions could have serious consequences for gene transcription/silencing and chromatin arrangement. Effects on transcription (via ubiquitylated histones) are thus a predicted response to proteotoxic-mediated effects on the ubiquitin cycle [40,41]. In addition, ubiquitylation of histones is involved in the regulation of other post-transcriptional histone modifications like acetylation and methylation [4]. Although histone ubiquitylation is a prerequisite for these modifications, they do not follow the same kinetics. Ubiquitin modified histones turn over every 2–3 hours [37] whereas acetylation and methylation modifications are much more stable [17,46]. Changes in the ubiquitin equilibrium are expected to influence these modifications translating in effects on transcription regulation and chromatin remodelling. These findings may point towards a ubiquitin-dependent regulation mechanism based on a delicate ubiquitin homeostasis. Consequently, different cellular processes can influence each other through the availability of the rate-limiting pool of free ubiquitin. This limited pool of free ubiquitin can be of functional significance to couple proteasomal activity to chromatin remodelling and in fact act as a novel signal transduction pathway, going from stress to signalling to the nucleus by affecting ubiquitin-histone modifications. Histone ubiquitylation is also important for other processes like DNA replication, repair and recombination. Often specific histones (like H2A, H3 and H4 for nucleotide excision repair) are targeted by ubiquitylation [20-22]. The key factor in the ubiquitin cycle is the existence of a limited pool of free ubiquitin, which couples the use of ubiquitin for poly-ubiquitin to (swift) effects on histone ubiquitylation. As a result, transcription, DNA repair and replication may all be affected by proteotoxic stress conditions.

## Conclusion and relevance

Regulated protein turnover by the ubiquitin-proteasome system (UPS) is essential for the survival of eukaryotic cells. This process is required for various cellular processes such as cell cycle control, signalling pathways, transcription and protein quality control. Alterations in the UPS are correlated with a variety of human pathologies, like cancer, immunological disorders, inflammation and neurodegenerative diseases [47]. The exact role of the UPS in the pathophysiology of these diseases however, remains poorly understood. Numerous studies suggest that inhibition of the proteasome may be efficient in the treatment in cancer and inflammation (reviewed in [48,49]). It is well established that many cancer cells are sensitive to proteasomal inhibitors, which often induce growth arrest and killing. Proteasome inhibitors will prevent the degradation of the regulator proteins resulting in cell cycle arrest and apoptosis. However, a disturbed ubiquitin homeostasis may contribute to cell death in proteasome inhibitor-treated cells as well. The pool of free ubiquitin can be depleted through capture in poly-ubiquitylated proteasomal substrates so that other ubiquitin-dependent processes are negatively affected. In addition, histone deubiquitylation may suffice to induce growth arrest.

Many neurological disorders such as Alzheimer's disease, Parkinson's disease, and Huntington's disease are caused by an accumulation of aberrant proteins leading to the formation of protein aggregates, inclusions and plaques. It is not completely clear why the UPS is failing to clear these aberrant proteins. For polyglutamine diseases like Huntington's disease it has been demonstrated that the UPS is unable to clear inclusions [50-52], and that proteasomes cannot degrade aggregated polyglutamine proteins [53] and polyglutamine peptides [54]. In some disorders, mutations in proteins of the UPS are implicated [55]. In addition to the accumulation of aberrant proteins many other abnormalities such as impaired axonal transport [56,57] and altered transcription regulation [58-61] are associated with these diseases (reviewed in [62]). Although these alterations in axonal transport and transcription regulation can be explained by interference of mutations in disease-related proteins, the pathogenic mechanisms leading to neuronal death and the involvement of protein aggregates are still largely unknown. Intriguingly, the processes that are affected in these disorders have at least one factor in common; they all require ubiquitin. In a number of disorders the accumulated proteins are ubiquitylated, while protein aggregates are also enriched in proteasomes. The question arises whether the sensitive ubiquitin equilibrium in these disorders is disturbed as a consequence of capture of poly-ubiquitylated proteins and/or inactive proteasomes in aggregates. A disturbed ubiquitin homeostasis might also contribute to alterations in, at first glance, unrelated cellular processes in neurological disorders. It is tempting to speculate that accumulation of aberrant proteins in these disorders disrupts the sensitive ubiquitin equilibrium, by trapping a significant fraction of ubiquitin and/or rendering proteasomes inactive in inclusion bodies and aggregates. As a consequence other processes requiring the availability of ubiquitin may be negatively influenced.

The flux of free ubiquitin between different cellular processes could be a passive mechanism in which unconjugated ubiquitin diffuses intracellular until it is utilized in ubiquitylation processes. However, a recent publication suggest that active factors could actually be involved in channelling ubiquitin from one ubiquitin-dependent process to another with temporally a higher priority [63]. It has been proposed that the DUB Doa1 helps to control DNA damage responses by releasing ubiquitin from proteasomal degradation into mechanisms involved in chromatin remodelling and DNA repair [63].

Ubiquitin seems more then just a signalling molecule involved in the regulation of various distinct processes in eukaryotic cells. The dynamic behaviour of ubiquitin modifications creates an equilibrium which allows crosstalk between different cellular processes that may allow cells to translate cellular stress to molecular responses by affecting the transcriptome.

## Abbreviations

DUBs, deubiquitylation enzymes; uH2A, ubiquitylated H2A; UPS, ubiquitin proteasome system.

## Competing interests

The author(s) declare that they have no competing interests.

## Authors' contributions

FAS and TAMG conceived the manuscript. JN and NPD revised the manuscript. All authors approved the final version.
